# Depression and anxiety symptoms in cardiac patients: a cross-sectional hospital-based study in a Palestinian population

**DOI:** 10.1186/s12889-019-6561-3

**Published:** 2019-02-26

**Authors:** H. Allabadi, A. Alkaiyat, A. Alkhayyat, A. Hammoudi, H. Odeh, J. Shtayeh, M. Taha, C. Schindler, E. Zemp, S. Haj-Yahia, N. Probst-Hensch

**Affiliations:** 1 Department of Epidemiology and Public Health, Swiss Tropical and Public Health Institute, Socinstrasse 57, P.O. Box, 4002 Basel, Switzerland; 2 University of Basel, Petersplatz 1, 4001 Basel, Switzerland; 3 Faculty of Medicine and Health Sciences, An-Najah National University, Rafidia Street, P.O. Box 7, Nablus, Palestine; 4 An-Najah National University Hospital, Asira Street, Nablus, Palestine; 5 School of Clinical Sciences, University of Bristol, 69 St Michael’s Hill, Bristol, BS2 8DZ UK; 6 Institute of Cardiovascular and Medical Sciences, Glasgow University, 126 University Place, Glasgow, G12 8TA UK

**Keywords:** Depression, Anxiety, Cardiovascular diseases, Predictors, Prevalence, Cardiac rehabilitation

## Abstract

**Background:**

Mental health problems have an adverse effect on the course of cardiac disease. The integration of their diagnosis and treatment into cardiology care is generally poor. It is particularly challenging in cultural environments where mental health problems are stigmatized. The objective of the current study was to investigate the proportion of cardiac patients with depression and anxiety as well as factors associated with the presence of these symptoms in a Palestinian population.

**Methods:**

This cross-sectional hospital-based study was conducted on patients consecutively admitted with a new or existing cardiac diagnosis to one of the four main hospitals in Nablus, Palestine over an eight-month period. Data was obtained from hospital medical charts and an in-person interview, using a structured questionnaire with a sequence of validated instruments. All subjects were screened for depression and anxiety using the Cardiac Depression Scale (CDS) and the Depression Anxiety Stress Scale (DASS-42). Multivariate ordered logistic regression analyses were performed to identify factors among four categories (socio-demographic, clinical, psychosocial, lifestyle) independently associated with depression and anxiety.

**Results:**

In total, 1053 patients with a confirmed cardiac diagnosis were included in the study with a participation rate of 96%. Based on the CDS and DASS-42, 54% met the criteria for severe depression (CDS > 100) and 19.2% for severe-to-very severe anxiety (DASS-anxiety > 15), respectively. Symptoms of depression and anxiety were more prevalent among females and less educated patients. Factors independently associated with both depressive and anxiety symptoms were post-traumatic stress disorder symptoms, low level of self-esteem, high somatic symptoms, low physical and mental health component scores, active smoking, physical inactivity, and longer disease duration. Patients with depressive and anxiety symptoms also reported poor social support and lower resilience.

**Conclusion:**

There was a high level of depression and anxiety in this sample of cardiac patients. The results point to characteristics of patients in particular need for mental health screening and suggest possible targets for intervention such as strengthening of social support and of physical activity. The integration of mental health services into cardiac rehabilitation in Palestine and comparable cultural settings is warranted from the time of first diagnosis and onward.

**Electronic supplementary material:**

The online version of this article (10.1186/s12889-019-6561-3) contains supplementary material, which is available to authorized users.

## Background

Cardiovascular diseases (CVDs) and depression are among the leading causes of the global disease burden [1]. CVDs are the most common cause of death accounting for 17.9 million deaths globally, in 2015 [2]. Depression affects over 300 million people around the world [3], and is expected to become the main cause of disability globally, in 2030 [4]. Similarly, in 2010, anxiety affected approximately 272 million people, worldwide [5].

There is a high prevalence of mental disorders, particularly depression and anxiety, in CVD patients [6]. The bidirectional link between CVDs and these mental disorders has been extensively documented in literature [7–9]. Approximately, 15–30% of patients with CVD suffer from depressive disorders [10–14]. These rates of depression are two to three times higher than in the general population [15]. Moreover, depression and anxiety have been found to worsen prognosis and quality of life in patients with coronary artery disease (CAD), myocardial infarction (MI), heart failure (HF), unstable angina, and coronary artery bypass grafting (CABG) [16–22]. CABG is defined as a surgical procedure that is performed to treat people who have severe coronary heart disease, which improves blood flow to the heart [23]. They were also found to be the biggest driver of health care costs in coronary heart disease (CHD) patients [24].

Mental health problems have a direct physiological effect on the course of cardiac disease and their adverse effect may be mediated by non-compliance to lifestyle interventions, treatment and medication [25, 26]. Furthermore, these mental disorders add to the burden of managing CVD, from a perspective of treatment complexity and emotional distress. This problem is further aggravated by the high rate of additional co-morbidities such as diabetes, hypertension and obesity [27].

The American Heart Association (AHA) has recommended routine depression screening in cardiac patients [26]. However, health systems have not yet adequately responded and less than 15% of cardiac patients are being diagnosed and treated for depression [28]. Integrating mental health into cardiac treatment is of particular relevance in low-middle-income countries (LMICs), where the burden of depression and anxiety is often high and aggravated by adverse life and political conditions. Similarly, mental health problems are stigmatized in many of these non-western cultures [29]. Little is known on the prevalence of depression and anxiety among cardiac patients in LMICs [30]. This also applies to Palestine, where mental disorders commonly are not recognized, diagnosed or treated, despite the increase in their prevalence [31]. Unaddressed mental care needs may be an important barrier to the successful management of cardiac patients in Palestine, where CVD remains the leading cause of death [32].

Mental health problems have not been studied in cardiac patients in the Palestinian population. Therefore, the aim of the current study was to determine the proportion of patients with depression and anxiety symptoms admitted with a cardiac diagnosis, to one of the four main hospitals in Nablus, Palestine. To guide physicians in effective mental health screening in the future, we also identified socio-demographic, clinical, psychosocial, and lifestyle factors associated with a high risk of depression and anxiety symptoms.

## Methods

### Study design and population

This cross-sectional hospital-based study was conducted on patients consecutively admitted to the cardiology and cardiac surgery departments of An-Najah National University Hospital, Arab-Specialized Hospital, Watani Hospital and Nablus Specialized Hospital in the Northern West Bank city of Nablus, Palestine. Patients were eligible for the study if they were between 30 and 80 years of age and had an existing or newly confirmed cardiac diagnosis warranting hospitalization during the period between March and November 2017. In the present study, cardiac diagnoses considered included CAD, ST elevation or non-ST elevation MI, angina, HF, cardiac arrhythmia, valve disease or any other cardiac disease. Diagnoses were confirmed using hospital medical charts. Patients were excluded if they had a normal cardiac catheterization (CATH), an acute or past stroke, end-stage kidney disease (including dialysis patients), peripheral vascular disease, major co-morbidities affecting mental health (alcohol abuse, drug abuse), neurological disorders (dementia, Alzheimer’s disease, epilepsy, Parkinson’s disease), cognitive impairment, a severe clinically diagnosed psychiatric condition or any other condition affecting the quality of their responses.

Eligible patients were recruited for in-person interviews by trained medical research assistants. Patients were identified using hospital registries and medical records. Interviews were conducted while patients were awaiting treatment (after CATH) or after their treatment, within 1 week of their admission to the hospital. Eligible patients were informed about the study objectives and benefits and written informed consent was obtained from those who agreed to participate. The study was approved by the Ethics Committee of Nordwest-und Zentral Schweiz (EKNZ) in Basel, Switzerland, and by the Institutional Review Board (IRB) committee at An-Najah National University in Nablus, Palestine.

### Study assessments and measures

Data was collected using a structured questionnaire consisting of two parts (Tables 1 and 2; see Additional file 1 for more detailed description). The first part included detailed socio-demographic and clinical information obtained from patients’ administrative and medical charts.

**Table 1:** Predictor blocks used in bivariate and ordered logistic regression analyses

Block	Variable
1 Socio-demographic factors	Age, gender, marital status, residence, education level, occupation
2 Clinical factors	Current diagnosis, previous cardiac diagnoses, years with cardiac disease, cardiac treatment (at admission), co-morbidities, medications, somatic symptoms (PHQ-15), family history of CVD, QoL (SF-12-PCS)
3 Psychosocial factors	PTSD (PTSD-PCL-S), social support (ESSI), resilience (RS-14), self-esteem, QoL (SF-12-MCS)
4 Lifestyle factors	Smoking status, currently on diet, fat consumption, vegetable and fruit consumption, alcohol use, physical activity, BMI

Note. *CVD* Cardiovascular disease, *QoL* Quality of life, *PHQ-15* Patient health questionnaire-15, *PCS* Physical component summary, *PTSD* Post-traumatic stress disorder, *PTSD-PCL-S* Post-traumatic stress disorder checklist, *ESSI* ENRICHD Social support instrument, *RS-14* Resilience scale-14, *MCS* Mental component summary, *BMI* Body mass index

**Table 2:** Study Instruments (see Additional file 1 for further descriptions)

Instrument	Description
Outcomes - assessment of depression, anxiety and stress	
Cardiac Depression (CDS)	The primary outcome of the study was measured using the Cardiac Depression Scale (CDS) by Hare et al., a disease-specific, 26-item questionnaire used to measure depression in patients with CVDs. CDS scores range from 26 to 182, and items are scored on a seven-point Likert scale from 1 (strongly disagree) to 7 (strongly agree) [60]. The CDS can be used as a continuous measure, where higher scores indicate higher depressive symptoms or as an ordinal indicator of possible depression using cut-off points previously used in literature. In this study, the presence of mild-to-moderate depression was defined as a CDS score of 90–100 and the presence of severe depression as a score >100. Scores below 90 indicated no-to-minimal depression [63].
Depression, anxiety, stress (DASS-42)	Depression (DASS-depression), anxiety (DASS-anxiety) and stress (DASS-stress) were measured using the Depression Anxiety Stress Scale-42 (DASS-42) by Lovibond & Lovibond, a 42-item questionnaire consisting of three subscales, each containing 14 items scored on a four-point Likert scale ranging from 0 (did not apply to me at all) to 3 (applied to me very much), measuring the extent to which each item was experienced over the past week. The scores are classified as: depression 0–9 (normal), 10–20 (mild-moderate), > 21 (severe-very severe); anxiety 0–7 (normal), 8–14 (mild-moderate), > 15 (severe-very severe); stress 0–14 (normal); 15–25 (mild-moderate), > 26 (severe-very severe) [64].
Predictors – correlates of depression and anxiety	
Somatic Symptoms (PHQ-15)	The Patient Health Questionnaire (PHQ-15) is a 15-item somatic symptom scale derived from the full Patient-Health-Questionnaire to measure the severity of somatization in patients [65].
Quality of life (SF-12-PCS; SF-12-MCS)	Quality of life was assessed using the 12-item Short-Form Health Survey (SF-12), which is a generic measure of overall health status. The SF-12 is comprised of two components, the Mental Component Summary (MCS) score and the Physical Component Summary (PCS) score [66].
Post-traumatic stress disorder (PTSD-PCL-S)	The Post-Traumatic Stress Disorder Checklist (PTSD-PCL-S) is a 17-item scale used to assess PTSD symptoms based on the Diagnostic and Statistical Manual of Mental Disorders (DSM IV) criteria. The PTSD-PCL-S is used to link symptom endorsements to a specific stressful or traumatic event or experience [67].
Social Support (ESSI)	The ENRICHD (Enhancing Recovery in Coronary Heart) Social Support Instrument (ESSI) is a seven-item scale comprised from the Medical Outcomes Survey (MOS). It assesses four components of social support including emotional, instrumental, informational and appraisal [68, 69].
Self-esteem (SE)	The Single-Item Self-Esteem Scale is a one-item scale developed as an alternative of the Rosenburg Self-Esteem scale [70].
Resilience (RS-14)	Resilience Scale (RS-14) is a 14-item questionnaire that assesses individual resilience in a general population [71].

The second part was administered during a private interview and consisted of a sequence of screening instruments. These validated tools have demonstrated to be suitable for clinical populations to assess for depression and anxiety symptoms, QoL, post-traumatic stress disorder (PTSD), social support, resilience, self-esteem, somatic symptoms and lifestyle behaviors. The questionnaire, including the instruments, was translated from English to Arabic and back-translated from Arabic to English by two bilingual experts.

### Statistical analyses

The primary endpoints in the present study were depression and anxiety. Descriptive statistics for stress as an endpoint are presented in an Additional file 2: Table S2, with no further description. The four predictor blocks investigated for association with depression and anxiety were socio-demographic, clinical, psychosocial and lifestyle factors (Table 1). Endpoints and predictors were described as means and standard deviations (SD) for quantitative variables and as absolute values and percentages for categorical variables. Differences in predictor variables according to presence or absence of depression and anxiety symptoms were described using chi-squared test and the Wilcoxon rank sum test, as appropriate. Fisher’s exact test was used for results presenting a frequency below five. Multivariate ordered logistic regression analyses were performed to examine the independent association between predictor variables and depression or anxiety symptoms. All variables were entered in each of the models at once. Results are presented in separate models for depression and anxiety and are expressed as odds ratio (OR) and 95% confidence intervals (95% CI). The cut-offs for the respective outcome variables were normal, mild/moderate, and severe/very severe according to standardized cut-offs. Correlations between the outcome variables and other instruments used in the study were assessed using Spearman’s rank correlation coefficient. Cronbach alpha was used to assess the internal consistency of the different scales. Statistical significance was defined as a two-sided *p*-value <0.05. All data was analyzed using the STATA Data Analysis and Statistical Software, version 14.

## Results

In total, 1092 patients were eligible and approached for an interview. Among the 1053 (96%) patients which agreed to participate in the study, 1022 patients with complete outcome and predictor information were used in the analysis.

### Characteristics of study population

Characteristics of the participants are presented in Table 3.

**Table 3:** Socio-demographic, clinical, psychosocial and lifestyle characteristics of study population, (*n* = 1022)

Variable	n(%)
Socio-demographic factors	
Age, mean (SD)	58.9 ± 10.1
Gender	
Female	272 (26.6)
Male	750 (73.4)
Marital status	
Married	926 (90.6)
Not married	96 (9.4)
Residence	
City	474 (46.4)
Village	473 (46.3)
Camp	75 (7.3)
Education degree	
No HS diploma	600 (58.7)
HS diploma	167 (16.3)
College degree	255 (25.0)
Occupation	
Professional	209 (20.5)
Non-professional	307 (30.0)
Unemployed	378 (37.0)
Retired	81 (7.9)
House wife	47 (4.6)
Clinical factors	
Cardiac diagnosis	
CAD	334 (32.7)
MI	406 (39.7)
Angina	162 (15.9)
Other	120 (11.7)
Previous cardiac diagnosis	
Yes	690 (67.5)
No	332 (32.5)
Years with cardiac disease	
≤ 1 year	628 (61.5)
2–9 years	255 (24.9)
≥ 10 years	139 (13.6)
Cardiac treatment (at admission)	
CATH/stent	534 (52.2)
CATH/CABG	240 (23.5)
CATH/other & unknown	248 (24.3)
Co-morbidities	
None	299 (29.3)
One	303 (29.6)
Two or more	420 (41.1)
Medications	
None	132 (12.9)
1–2	152 (14.9)
3–4	738 (72.2)
Somatic symptoms (PHQ-15)	
Minimal	91 (8.9)
Low	241 (23.6)
Medium	314 (30.7)
High	376 (36.8)
Family history	
Yes	614 (60.1)
No	408 (39.9)
QoL, (SF-12-PCS score), mean (SD)	37.6 ± 12.4
Psychosocial factors	
PTSD (PTSD-PCL-S)	
Minimal	611 (59.7)
Some	95 (9.3)
Moderate	253 (24.8)
High	63 (6.2)
Social support (ESSI)	
Low	364 (35.6)
High	658 (64.4)
Resilience (RS)	
Very low	92 (9.0)
Low	103 (10.1)
Low-end	204 (20.0)
Moderate	257 (25.1)
Moderately-high	274 (26.8)
High	92 (9.0)
Self-esteem (SE) score, mean (SD)	5.8 ± 1.4
QoL,(SF-12-MCS score), mean (SD)	39.7 ± 13.2
Lifestyle factors	
Smoking status	
Never	364 (35.6)
Former	170 (16.6)
Current	488 (47.8)
Currently on diet	
Yes	170 (16.6)
No	852 (83.4)
Fat consumption	
Low	480 (47.0)
Medium	323 (31.6)
High	219 (21.4)
Vegetable & fruit consumption	
Low	102 (10.0)
Medium	353 (34.5)
High	567 (55.5)
Alcohol use, (*n* = 1019)	
Yes	49 (4.8)
No	973 (95.2)
Physical activity	
None	337 (33.0)
Not daily	189 (18.5)
Daily	496 (48.5)

Note. *HS* High school, *MI* Myocardial infarction, *CAD* Coronary artery disease, *CATH* Catheterization, *CABG* Coronary artery bypass graft, *CVD* Cardiovascular disease, *PHQ-15* Patient health questionnaire-15, *PCS* Physical component summary, *QoL* Quality of life, *SD* Standard deviation, *PTSD* Post-traumatic stress disorder, *PTSD-PCL-S* Post-traumatic stress disorder checklist, *ESSI* ENRICHD social support instrument, *RS-14* Resilience scale-14, *MCS* Mental component summary, *BMI* Body mass index

#### Socio-demographic factors

Among the 1022 patients, 73.4% were males. The mean age of patients was 58.9 ± 10.1 years (range 30–80 years). The majority of participants were married (90.6%), 37% were unemployed, and 58.7% did not have a high school diploma. Most of the study population lived in cities (46.4%) or villages (46.3%), while 7.3% resided in refugee camps.

#### Clinical factors

The primary diagnoses among the sample were MI (39.7%), CAD (32.7%), angina (15.9%) and other diagnoses (11.7%), including mitral or aortic valve stenosis, valve regurgitation, heart block and others. Among the category of other diagnoses, 29 (2.8%) patients had heart failure. Over 60% of participants had a previous cardiac diagnosis and had been diagnosed with a cardiac disease for 1 year or less. About half of participants underwent a CATH with a stent (52.2%), while others underwent a CATH with a CABG (23.5%), or some kind of other procedure (24.3%), which was not yet performed at the time of the interview. Forty one percent of participants reported having two or more co-morbidities (mainly diabetes and hypertension) and 72.2% were on three or more medications. In addition, more than half of the participants’ reported a family history of CVD. Approximately 37% of participating patients exhibited high somatic symptoms on the PHQ-15. The mean score of the participants on the SF-12- PCS score was 37.6 ± 12.4.

#### Psychosocial factors

Forty Percent of patients reported having PTSD symptoms on the PTSD-PCL-S. Social support was generally high (64%) among participants, according to the ESSI. Almost half of the participants presented with moderate to moderately-high resilience. The mean score for self-esteem was 5.8 ± 1.4, while the mean score for the SF-12-MCS was 39.7 ± 13.2.

#### Lifestyle factors

Almost half of the participants were current smokers while 35.6% had never smoked before. The vast majority of participants (83.4%) reported not being on a diet, 47% low fat consumption, and 55.5% high vegetable and fruit consumption. The reported alcohol consumption was very low with 95% of participants not consuming any alcohol. Almost half of the patients reported no-to-minimal daily physical activity. Eighty percent of patients were either overweight or obese.

### Proportion of patients with depressive and anxiety symptoms at different severity levels

Table 4 shows the proportion of patients with depression (CDS), depression (DASS-depression), anxiety (DASS-anxiety) and stress (DASS-stress) symptoms at different severity levels. Cutoffs for the levels of CDS and DASS subscales are also presented in Table 4. Based on our findings, the mean ± SD depression score on the CDS was 101.3 ± 15.6 and the overall proportion of patients with depression was 78.7%. According to the recommended cutoffs for the CDS, 21.3, 25.2 and 53.5% of the sample had no, mild-to-moderate and severe-to-very severe depression symptoms, respectively. The means ± SDs on the DASS-42 were 9.4 ± 8.6, 9.4 ± 6.8 and 15.2 ± 9 for depression, anxiety, and stress, respectively. It was found that the overall proportion was 52.9, 53.1 and 37.4% for the presence of depressive, anxiety and stress symptoms according to the DASS-42. Based on recommended cutoffs, 47.1, 33.4 and 19.5% of patients reported normal, mild-to-moderate and severe-to-very severe depressive symptoms (DASS-depression). In addition, 46.9% patients did not report any anxiety, while 33.9% reported mild-to-moderate anxiety and 19.2% reported severe-to-very severe anxiety symptoms. According to the stress scale, 62.6% reported having no stress, 30% reported having mild-moderate stress symptoms and 7.4% severe-very severe stress symptoms. Patients that had mild-moderate and severe-very severe symptoms of depression or anxiety according to the DASS-42 were more likely to have severe depressive symptoms on the CDS. Nevertheless, 8 (4.0%) and 9 (4.6%) patients without any signs of depressive symptoms on the CDS, showed symptoms of depression and anxiety, respectively, on the DASS-42.

**Table 4:** Proportion of patients with CDS-depression, DASS-depression, DASS-anxiety, DASS-stress at different severity levels (*n* = 1022)

	Depression, anxiety, stress according to CDS levels			
	Normal n (%)	Mild- Moderate n (%)	Severe-very severe n (%)	Percentage above normal level
CDS	CDS < 90 218 (21.3)	CDS 90–100 257 (25.2)	CDS > 100 547 (53.5)	78.7
DASS-depression (D ≥ 10)	481 (47.1)	341 (33.4)	200 (19.5)	52.9
Normal (0–9)	166 (34.5)	164 (35.1)	151 (31.4)	
Mild/moderate (10–20)	44 (12.9)	82 (24.1)	215 (63.1)	
Severe/very severe (21–42)	8 (4.0)	11 (5.5)	181 (90.5)	
DASS-anxiety, (A ≥ 7)	479 (46.9)	347 (33.9)	196 (19.2)	53.1
Normal (0–6)	152 (31.7)	164 (34.2)	163 (34.0)	
Mild/moderate (7–14)	57 (16.4)	77 (22.2)	213 (61.4)	
Severe/very severe (15–42)	9 (4.6)	16 (8.2)	171 (87.2)	
DASS-stress, (S ≥ 15)	640 (62.6)	307 (30.0)	75 (7.4)	37.4
Normal (0–14)	178 (27.8)	206 (32.2)	256 (40.0)	
Mild/moderate (15–25)	36 (11.7)	44 (14.3)	227 (73.9)	
Severe/very severe (26–42)	4 (5.3)	7 (9.3)	64 (85.3)	

Note. *CDS* Cardiac depression scale, *DASS* Depression, anxiety, stress scale

### Unadjusted correlations between continuous scores of the scales used in study

**Fig. 1:**
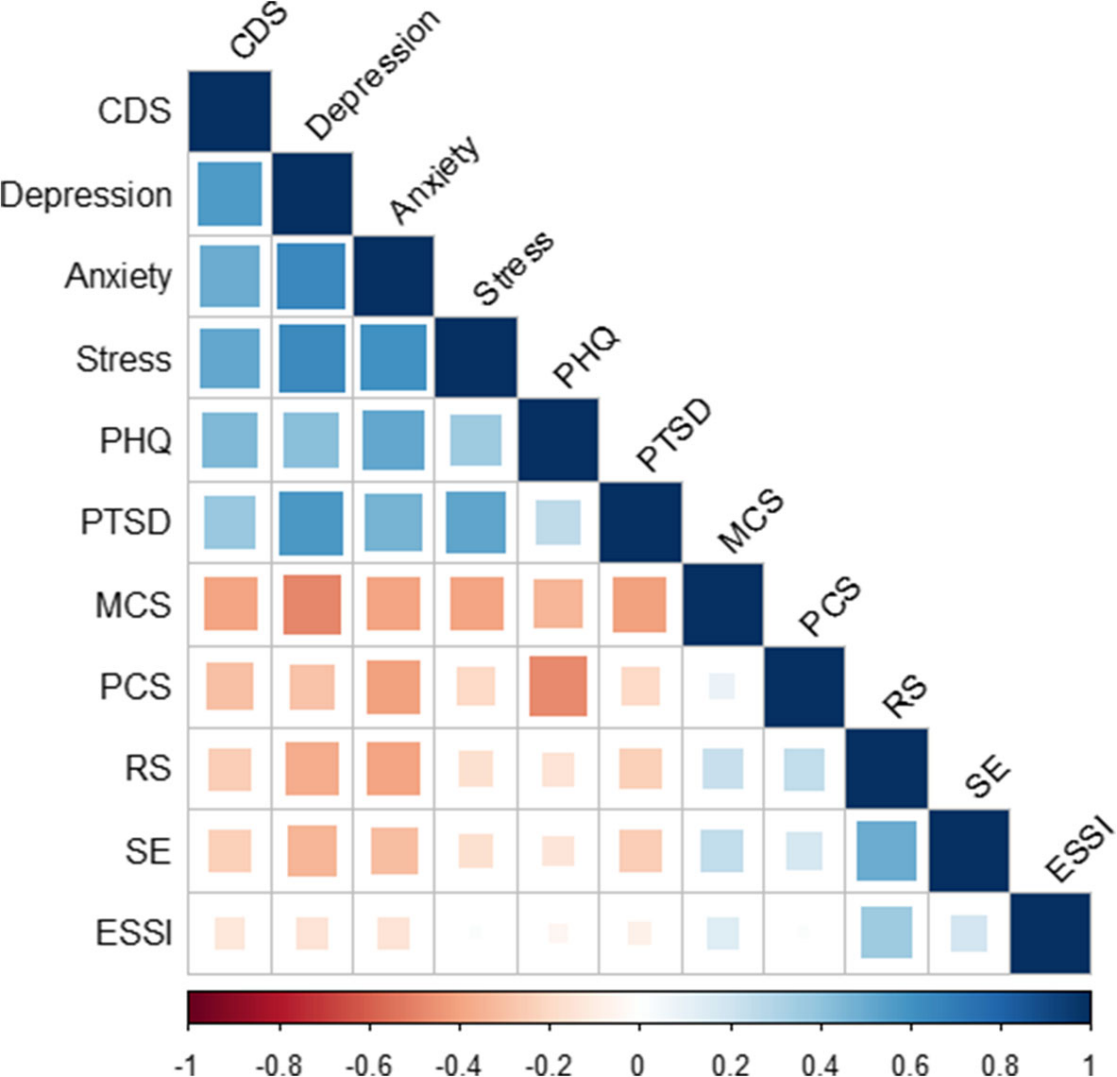
Spearmen correlations between CDS, DASS-depression, DASS-anxiety, DASS-stress and other instruments used in the study. Note. CDS = Cardiac Depression Scale; PHQ = Patient Health Questionnaire-15; PTSD = Post-Traumatic Stress Disorder; MCS = Mental Component Summary; PCS = Physical Component Summary; RS = Resilience Scale-14; SE = Self-esteem; ESSI = ENRICHD Social Support Instrument. Blue colors represent positive correlations and red colors represent negative correlations. Correlations measured are expressed as rho spearman.

Spearman correlation coefficients were calculated between the CDS and other instruments used in the study (see Fig. 1). The correlation of the CDS with the DASS-depression was =0.57 (p = < 0.001), with DASS-stress =0.51 (*p* < 0.001), and with DASS-anxiety =0.50 (p < 0.001). The correlations between the DASS subscales were as follows: DASS-depression and DASS-anxiety (0.65, p < 0.001); DASS-depression and DASS-stress (0.63, p < 0.001); DASS-anxiety and DASS-stress (0.61, p < 0.001). CDS was positively correlated with the PHQ-15 (0.44, *p* < .001) and the PTSD-PCL-S (0.37, p < .001)and the DASS-depression was also positively correlated with the PTSD-PCL-S (0.57, p < .001).

The above cluster of positively associated factors were weakly correlated to the physical and mental QoL components of the SF-12, social support, resilience, and self-esteem, which were strongly correlated among each other. Specifically, weak correlations were observed between CDS and the SF-12-MCS (− 0.39, p < 0.001), SF-12-PCS (− 0.29, p < 0.001), ESSI (− 0.12 p < 0.001), RS-14 (− 0.24, p < 0.001) and with the self-esteem scale (− 0.23, *p* < 0.001).

### Association between socio-demographic, clinical, psychosocial, lifestyle factors and depressive and anxiety symptoms

The bivariate distribution of socio-demographic, clinical, psychological, and lifestyle characteristics according to the presence or absence of depression symptoms (CDS), anxiety symptoms (DASS-anxiety) and stress symptoms (DASS-stress) is presented in Additional file 3: Table S1 (depression (CDS) and anxiety) and Additional file 2: Table S2 (stress). Briefly, with regard to socio-demographic factors, bivariate analysis revealed depressive and anxiety symptoms were more frequent among females than males (depression: 84.9% vs. 76.4%, *p* = 0.003; anxiety: 72.4% vs. 55.6%, p = < 0.001) and among those with lower educational level (depression: 81.0% [no high school diploma] vs. 78.0% [high school diploma] and 71.1% [college degree], *p* = 0.02; anxiety: 63.2% [no high school diploma] vs. 59.8% (high school diploma) and 52.8% (college degree), *p* < 0.05). Both, depressive and anxiety symptoms were most prevalent among those unemployed and housewives (depression: p < 0.001; anxiety: p < 0.001).

Multivariable ordered logistic regression was performed to determine the independent association of factors in the four predictor blocks with a) depressive symptoms, categorized as no depressive symptoms, moderate depressive symptoms, and severe depressive symptoms according to the CDS and b) anxiety symptoms, categorized as minimal anxiety symptoms, mild-moderate anxiety symptoms, and severe-severe anxiety symptoms according to the DASS-anxiety subscale. The results for the four blocks of variables are presented in Table 5.

**Table 5:** Factors associated with DEPRESSION and ANXIETY in multivariate ordered logistic regression

Variable	Depression (CDS)			Anxiety (DASS-anxiety)		
	OR	95% CI	*P* value	OR	95% CI	*P* value
Socio-demographic factors						
Age, mean (SD)	0.99	(0.98–1.00)	0.393	**0.96**	**(0.95–0.99)**	**< 0.001**
Gender						
Female	(reference)			(reference)		
Male	0.83	(0.51–1.36)	0.469	0.68	(0.43–1.10)	0.117
Marital status						
Married	(reference)			(reference)		
Not married	**0.38**	**(0.22–0.65)**	**0.001**	1.28	(0.76–2.13)	0.350
Residence						
City	(reference)			(reference)		
Village	1.22	(0.91–1.64)	0.191	0.74	(0.54–1.00)	0.054
Camp	1.35	(0.76–2.40)	0.308	0.56	(0.31–1.01)	0.054
Education degree						
No HS diploma	(reference)			(reference)		
HS diploma	1.15	(0.82–1.62)	0.421	0.93	(0.65–1.31)	0.666
College degree	1.19	(0.76–1.87)	0.449	1.09	(0.68–1.76)	0.715
Occupation						
Professional	(reference)			(reference)		
Non-professional	0.85	(0.55–1.31)	0.472	1.15	(0.73–1.81)	0.544
Unemployed	1.12	(0.67–1.88)	0.662	0.89	(0.54–1.48)	0.660
Retired	0.71	(0.38–1.33)	0.289	1.44	(0.75–2.73)	0.269
House wife	**0.33**	**(0.14–0.80)**	**0.014**	0.65	(0.27–1.56)	0.335
Clinical Factors						
Cardiac diagnosis						
CAD	0.89	(0.63–1.25)	0.508	0.75	(0.53–1.07)	0.111
MI	(reference)			(reference)		
Angina	1.26	(0.83–1.92)	0.271	0.54	(0.35–0.86)	0.009
Other	0.82	(0.49–1.37)	0.440	1.23	(0.76–1.99)	0.407
Previous cardiac diagnosis						
Yes	**0.67**	**(0.47–0.96)**	**0.028**	**1.52**	**(1.05–2.21)**	**0.026**
No	(reference)			(reference)		
Years with cardiac disease						
≤ 1 year	(reference)			(reference)		
2–9 years	**1.46**	**(1.00–2.13)**	**0.048**	1.10	(0.76–1.59)	0.663
≥ 10 years	**1.71**	**(1.06–2.75)**	**0.028**	0.95	(0.60–1.50)	0.832
Cardiac treatment (at admission)						
CATH/stent	(reference)			(reference)		
CATH/CABG	1.42	(0.98–2.04)	0.065	0.89	(0.62–1.29)	0.558
CATH/other & unknown	1.34	(0.95–1.89)	0.097	**1.44**	**(1.02–2.04)**	**0.036**
Co-morbidities						
None	(reference)			(reference)		
1	0.93	(0.64–1.34)	0.695	0.96	(0.65–1.43)	0.860
2+	1.39	(0.94–2.04)	0.096	1.36	(0.92–2.01)	0.127
Medications						
None	(reference)			(reference)		
1–2	1.33	(0.78–2.29)	0.293	1.20	(0.68–2.10)	0.521
3–4	1.19	(0.74–1.92)	0.470	1.31	(0.79–2.18)	0.299
Somatic symptoms (PHQ-15)						
Minimal	(reference)			(reference)		
Low	1.18	(0.72–1.94)	0.506	1.41	(0.74–2.72)	0.296
Medium	**1.86**	**(1.12–3.08)**	**0.015**	**3.35**	**(1.76–6.35)**	**< 0.001**
High	**3.00**	**(1.73–5.18)**	**< 0.001**	**7.64**	**(3.96–14.78)**	**< 0.001**
Family history						
Yes	1.03	(0.77–1.38)	0.824	0.76	(0.57–1.03)	0.075
No	(reference)			(reference)		
QoL, (SF-12-PCS score)	**0.98**	**(0.97–1.00)**	**0.015**	**0.97**	**(0.96–0.99)**	**< 0.001**
Psychosocial factors						
PTSD (PTSD-PCL-S)						
Minimal	(reference)			(reference)		
Some	1.32	(0.83–2.10)	0.247	**2.34**	**(1.47–3.71)**	**< 0.001**
Moderate	**1.87**	**(1.29–2.71)**	**0.001**	**3.01**	**(2.12–4.27)**	**< 0.001**
High	1.28	(0.00)	0.974	**7.37**	**(3.89–14.0)**	**< 0.001**
Social support (ESSI)						
Low	(reference)			(reference)		
High	**0.71**	**(0.52–0.97)**	**0.032**	**0.74**	**(0.54–1.00)**	**0.049**
Resilience (RS-14)						
Very low	(reference)			(reference)		
Low	1.05	(0.49–2.25)	0.896	0.76	(0.41–1.41)	0.381
Low-end	0.78	(0.39–1.56)	0.479	**0.43**	**(0.25–0.77)**	**0.004**
Moderate	0.60	(0.30–1.21)	0.154	**0.37**	**(0.20–0.66)**	**0.001**
Moderately-high	**0.48**	**(0.23–0.98)**	**0.044**	**0.19**	**(0.10–0.36)**	**< 0.001**
High	**0.42**	**(0.18–0.94)**	**0.035**	**0.22**	**(0.11–0.48)**	**< 0.001**
Self-esteem (SE) score	0.97	(0.85–1.10)	0.640	0.85	(0.76–0.96)	**0.007**
QoL,(SF-12-MCS score)	**0.96**	**(0.95–0.97)**	**< 0.001**	**0.98**	**(0.97–0.99)**	**< 0.001**
Lifestyle factors						
Smoking status						
Never	(reference)			(reference)		
Former	**1.87**	**(1.17–2.97)**	**0.008**	0.71	(0.44–1.14)	0.154
Current	**1.61**	**(1.11–2.33)**	**0.013**	1.37	(0.93–2.02)	0.106
Currently on diet						
Yes	**0.59**	**(0.40–0.86)**	**0.007**	1.45	(0.98–2.13)	0.060
No	(reference)			(reference)		
Fat consumption						
Low	(reference)			(reference)		
Medium	0.73	(0.53–1.00)	0.054	1.09	(0.79–1.51)	0.583
High	0.79	(0.55–1.14)	0.206	1.28	(0.88–1.86)	0.187
Vegetable & fruit consumption						
Low	(reference)			(reference)		
Medium	0.83	(0.50–1.37)	0.466	**0.52**	**(0.31–0.86)**	**0.011**
High	0.73	(0.45–1.19)	0.207	0.80	(0.49–1.30)	0.375
Alcohol use						
Yes	1.31	(0.66–2.61)	0.439	1.19	(0.63–2.24)	0.591
No	(reference)			(reference)		
Physical activity						
None	(reference)			(reference)		
Not daily	**0.64**	**(0.42–0.98)**	**0.040**	**0.57**	**(0.37–0.87)**	**0.009**
Daily	**0.43**	**(0.30–0.60)**	**< 0.001**	0.98	(0.70–1.38)	0.908
BMI						
Underweight	0.63	(0.05–7.7)	0.720	2.09	(0.22–19.9)	0.522
Normal weight	(reference)			(reference)		
Overweight	0.81	(0.55–1.18)	0.272	**0.61**	**(0.42–0.89)**	**0.010**
Obese	0.95	(0.63–1.43)	0.824	0.87	(0.59–1.29)	0.495

Note. Multivariate ordered logistic regression reported for socio-demographic, clinical, psychosocial and lifestyle factors associated with depression, CDS (not depressed; mild-moderate depression; severely-very severe depression) and DASS-anxiety (no anxiety, mild-moderate anxiety; severe-very severe anxiety). Analyses were performed in two separate models for depression and anxiety, mutually adjusting for all factors in the four predictor blocks in both models. Analyses are also adjusted for the hospital to which patients were admitted. *OR* Odds ratio, *CI* Confidence Interval, *SD* Standard deviation, *HS* High school, *MI* Myocardial infarction, *CAD* Coronary artery disease, *CATH* Catheterization, *CABG* Coronary artery bypass graft, *PHQ-15* Patient health questionnaire-15, *PCS* Physical component summary, *PTSD* Post-traumatic stress disorder, *PTSD-PCL-S* Post-traumatic stress disorder checklist, *ESSI* ENRICHD social support instrument, *RS-14* Resilience scale-14, *MCS* Mental component summary, *BMI* Body mass index; *P* values in bold are significant at *p* < 0.05

Overall, most of the psychosocial factors were consistently associated with both, depression and anxiety. Participants with depression or anxiety were more likely to exhibit at least some symptoms of PTSD. Odds ratios tended to be higher for anxiety than for depression (moderate symptoms vs. minimal symptoms: OR_depresssion_ 1.87 (95% CI 1.29–2.71) vs. OR_anxiety_ 3.01 (95% CI 2.12–4.27). Patients with depression or anxiety had a lower score for the mental component of QoL (SF-12-MCS) [OR_depression_ 0.96 (95% CI 0.95–0.97); OR_anxiety_ 0.98 (95% CI 0.97–0.99)]. High resilience and high social support were inversely associated with depression and anxiety [high vs. low social support: OR_depression_ 0.71 (95% CI 0.52–0.97); OR_anxiety_ 0.74 (95% CI 0.54–1.00)]. The inverse association with resilience tended to be stronger in the presence of anxiety [high vs. very low resilience: OR_depression_ 0.42 (95% CI 0.18–0.94); OR_anxiety_ 0.22 (95% CI 0.11–0.48)].

In addition to psychosocial factors, the physical component of QoL (SF-12-PCS) and somatic symptoms (PHQ-15) also showed consistent associations with both depression and anxiety [high vs. minimal somatic symptoms: OR_depression_ 3.00 (95% CI 1.73–5.18); OR_anxiety_ 7.64 (95% CI 3.96–14.78)]; [SF-12-PCS: OR_depression_ 0.98 (95% CI 0.97–1.00); OR_anxiety_ 0.97 (95% CI 0.96–0.99)]. Current smoking was associated with depression with the strongest odds ratio observed for depression and former smoking: OR 1.87 (95% CI 1.17–2.97). Finally, patients with depression and anxiety were more likely to be physically inactive compared to patients without the respective psychological problems.

A few factors exhibited associations with only one of the two mental health outcomes. For example, patients residing in villages or camps were less likely to show symptoms of anxiety compared to patients living in the city [(OR_village_ 0.74 (0.54–1.00); OR_camp_ 0.56 (0.31–1.01)]. Unemployment was positively associated with depression, but not anxiety. The presence of previous cardiac diagnoses was positively associated with anxiety, but inversely associated with depression [(OR_depression_ 0.67 (95% 0.47–0.96); OR_anxiety_ 1.52 (95% 1.05–2.21)]. Symptoms of depression were more frequent among patients with a cardiac diagnosis for more than 10 years compared to patients with a diagnosis for a year or less [years since first diagnosis ≥10 vs. ≤ 1 year OR 1.71 (95% 1.06–2.75)]. Patients who were diagnosed with angina were more likely to have anxiety symptoms than those diagnosed with an MI. Underweight and obese participants were more likely to exhibit symptoms of anxiety than those of normal weight and overweight.

### Reliability assessment (Cronbach’s alpha) of the study instruments

Cronbach α for the primary outcome variable, CDS was 0.86 and 0.92 (DASS-depression), 0.82 (DASS-anxiety), and 0.89 (DASS-stress) for the DASS subscales, respectively, indicating high consistency for the relevant psychometric scales. This indicates all scales exhibit acceptable internal consistency with little likelihood of item redundancy. Inter-item correlations between the 26 items of the CDS and the total CDS scores ranged from 0.08 to 0.58, and all correlations were statistically significant at the 0.01 level. Cronbach α for the other scales used in the study was: 0.78 for PHQ-15, 0.86 for PTSD-PCL-S, 0.82 for ESSI and 0.88 for RS-14.

## Discussion

In the present study, the observed rates of depressive and anxiety symptoms were high. Only 21% (CDS) and 46% (DASS-anxiety) of patients did not exhibit any symptoms of depression and anxiety, respectively. Our findings point to the need for integrating mental health care into cardiac treatment. It is noteworthy that several factors found to be associated with depression and anxiety may serve as screening and possibly as intervention targets.

Rates of mental health problems reported in earlier studies for patients with different cardiac diagnoses and in different cultural and health system settings ranged from 14 to 73% [33–38] for depressive symptoms and 15 to 48% [33–37] for anxiety symptoms. These varying rates are explained in part by differences in sample sizes, the instruments and cutoffs used for classifying depression and anxiety and the type of cardiac disease targeted in studies.

Lower rates of depression than in the current study were observed in other settings including Norway (14%) [33], USA (15%) [39], Brazil (26.4%) [34] and Pakistan (14%) [36]. Similar among these studies, was the common psychiatric instruments used to assess for depression, all of which were not specific for cardiac populations. In contrast, a different study assessing depression using the CDS found a rate of 73.2% of severe depression in Iranian patients with acute coronary syndrome (ACS), a rate even higher than in this study [38].

Similar anxiety rates to the current study were observed in Iran (28.5%) [37] and Pakistan (18%) [36]. Interestingly, in another study conducted in Brazil, Meneghetti et al. found a very high prevalence of 48.4% for anxiety symptoms among ACS patients using the Hospital Anxiety and Depression Scale (HADS) [34]. A study in the USA also reported, 37% of patients with MI due to spontaneous coronary artery dissection screened for anxiety using the Generalized Anxiety Disorder 7-Item Scale (GAD-7) [35].

Mental health problems are generally high in the Palestinian population [31]. In the absence of a healthy control group the results of this study do not allow to conclude that depression and anxiety are more common in cardiac patients. However, cardiac patients are in particular need of treatment for depression and anxiety given that existing evidence points to their adverse effect on the course of heart disease. Furthermore, cardiac rehabilitation may be an efficient starting point to address mental health issues beyond the patient and to the extended family and social network. Given the shortage of mental health services available and the local economic instability in Palestine, the provision of additional services needs to be implemented in a cost-effective way. The identification of subgroups of cardiac patients at higher risk of depression and anxiety can guide screening and interventions.

In perspective of mental health screening among cardiac patients, focus should be given to females and less educated patients. The higher rates of depression and anxiety seen in these sub-groups were previously described in literature [34, 37, 40, 41]. Women seem to be more vulnerable to the trauma caused by cardiac events, which leads to a deterioration in depression and anxiety symptoms [37]. As observed in some [34, 42, 43], but not all studies [37, 38, 44], the association between gender and social status may not be direct as suggested by the disappearance of gender and social status differences in the fully adjusted models [34, 37, 38, 42].

The presence of the following additional characteristics in cardiac patients should be a red flag for cardiologists to consider mental health care in cardiac practice: symptoms of PTSD, low levels of self-esteem, somatic symptoms, low QoL components, active smoking, physical inactivity, and longer disease duration. In contrast, a high level of resilience seems to reduce symptoms of psychological problems, as previously observed in patients with heart failure [45, 46]. Unlike findings reported previously, comorbidities were not consistently more common in the presence of mental health disorders [47]. Little is known about the association of PSTD symptoms with depression and anxiety in cardiac patients. A study conducted on 813 patients who received angiograms at a large U.S. Veterans Administration Medical Center found depression to be positively associated with PTSD, smoking and alcohol consumption [48]. Low MCS and PCS scores on the SF-12, smoking, and chest pain were recently identified as the strongest predictors of longitudinally sustained high levels of depression and anxiety in CHD patients [24].

Factors serving as targets for intervention include smoking, physical activity and social support. Smoking cessation interventions are crucial for cardiac rehabilitation, however in the presence of depression, results are less successful and interventions may need to be adapted [49]. Sedentary behavior, a risk factor for depression in the general population [50], was previously associated with depression according to the Beck Depression Inventory-II in patients hospitalized for ACS [51]. In a small non-randomized intervention study with heart failure patients, aerobic interval training decreased symptoms of depression over a period of 12-weeks [52]. In studies on breast cancer [53], promotion of physical activity may have the additional benefit of improving self-esteem, a factor associated with depression and anxiety in this study and a predictor of mortality in the general population [54]. In addition, according to previous studies [43, 55–58], the inverse association between high social support and low levels of anxiety and depression points to another important target for prevention as it is supported by firm evidence from previous studies. Poor social support among patients with ACS was observed in secondary analyses of a randomized trial to reduce the effectiveness of treatment with antidepressants [59]. The quality of social support plays an important role, as overprotective behaviors of partners can have an adverse effect [58]. Interestingly, in the current study marital status and social support were associated with presence of depressive symptoms independently and in opposite directions.

The current study has several strengths. First, it utilized a broad set of validated instruments to identify depression and anxiety symptoms as well as associated factors. The overall validity of the CDS in this study was almost similar to levels originally reported by Hare and Davis [60]. The validity of the DASS-42 was satisfactory, in line with other findings of other studies, including those originally reported by Lovibond and Lovibond [61]. Furthermore, the CDS is the only psychometric scale suitable for the comparative depression assessment in heart disease patients, subjected to different interventions [60, 62] . This is evident in the present study, as depression rates were lower when assessed by the DASS-42. The CDS, also has excellent properties for the diagnosis of MDD, a score of ≥95 having a 97% sensitivity at 85% specificity (AUC 0.96) [62]. Second, the large sample size provided sufficient statistical power for testing independent associations. Third, the study subjects are well characterized, which allowed for addressing confounding. Fourth, the high participation rate decreased the likelihood of selection and participation bias. Finally, the findings of the study despite being hospital-based are likely generalizable to the entire cardiac patient population in the city of Nablus and surrounding cities of Palestine, considering the study sites provide cardiac care to a large percentage of cardiac patients in the area.

Nonetheless, the cross-sectional nature of the study does not enable us to make causal inferences. A long-term follow-up is foreseen to investigate the predictive effect of the study characteristics and predictors with regard to the course of depression, anxiety and heart disease. While the reliability of the previously validated instruments was confirmed in our study, the instruments have not been validated specifically for the context of cardiac patients in Palestine. Furthermore, some of the risk factors could only be captured broadly to avoid lengthy interviews. This likely caused some misclassification, as in the case of physical activity. Recall bias may have added to the misclassification of risks, given the retrospective nature of the interview. Also, the study was not sufficiently powered to investigate differences in the frequency of depression and anxiety as well as associated factors between relevant subgroups of clinical diagnoses, for example MI and HF. Finally, some patients were recruited at the time of admission and for the most part before receiving their intervention or diagnosis, and thus were under much pressure and stress. The level of depression and anxiety could have been overestimated and may decrease in part over the course of disease. The most appropriate time point to assess depression and anxiety from a prognostic perspective is unknown.

## Conclusions

The alarmingly high rate of depression and anxiety symptoms observed in cardiac patients in Palestine points to the need for integrating mental health care into cardiac rehabilitation. The prognostic value of depression and anxiety with regard to the course of heart disease, adherence to treatment and quality of life needs to be investigated. Treatment of psychological problems from the disease onset and onwards is crucial considering longer disease duration puts individuals at higher risk of being depressed. The expertise of social scientists and medical anthropologists is needed for identifying efficient means to overcome barriers related to the stigmatization of psychological disorders.

## Additional files

Additional file 1: Description of study instruments. (DOCX 39 kb)

Additional file 2: **Table S2.** Socio-demographic, clinical, psychosocial, lifestyle factors by STRESS status, (n = 1022). (DOCX 24 kb)

Additional file 3: **Table S1.** Socio-demographic, clinical, psychosocial, lifestyle factors by DEPRESSION and ANXIETY status, (*n* = 1022). (DOCX 24 kb)

## Abbreviations

ACS: Acute coronary syndrome
AHA: American Heart Association
BMI: Body mass index
CABG: Coronary artery bypass grafting
CAD: Coronary artery disease
CATH: Catheterization
CDS: Cardiac Depression Scale
CHD: Coronary heart disease
CI: Confidence interval
CVD: Cardiovascular disease
CVDs: Cardiovascular diseases
DASS-42: Depression Anxiety Stress Scale-42
DSM-IV: Diagnostic and Statistical Manual of Mental Disorders-IV
EKNZ: Ethics Committee of Nordwest-und Zentral Schweiz
ENRICHD: Enhancing Recovery in Coronary Heart
ESSI: ENRICHD Social Support Instrument
HF: Heart failure
HS: High school
IRB: Institutional Review Board
LMIC: Low-middle income countries
MCS: Mental Component Summary
MDD: Major depressive disorder
MI: Myocardial infarction
MOS: Medical Outcomes Survey
OR: Odds ratio
PCS: Physical Component Summary
PHQ-15: Patient Health Questionnaire-15
PTSD: Post-traumatic stress disorder
PTSD-PCL-S: Post-Traumatic Stress Disorder Checklist
QOL: Quality of life
RS-14: Resilience Scale-14
SD: Standard deviation
SE: Self-esteem
SF-12: The 12-item Short Form Health Survey
