# Supplementary material for: Depression and anxiety symptoms in cardiac patients: a cross-sectional hospital-based study in a Palestinian population
Source: BMC Public Health. 2019 Feb 26;19:232. doi: 10.1186/s12889-019-6561-3 (PMC6390372; doi:10.1186/s12889-019-6561-3)
Supplement: Supplementary file 1 — Description of study instruments. (DOCX 39 kb) [file 12889_2019_6561_MOESM1_ESM.docx]

**Additional file: Description of study instruments**

***Cardiac Depression (CDS).*** The Cardiac Depression Scale (CDS), by Hare et al., is a 26-item questionnaire used to measure depression in patients with CVD [1]. It has been used and validated in cardiac patients with a range of diagnoses such as HF, post-MI, CAD, and arrhythmias. The CDS has accurate psychometric properties including internal and external reliability and validity. Items on the scale are measured on a seven-point Likert scale from 1 (strongly disagree) to 7 (strongly agree). CDS scores range from 26-182. The CDS can be used as a continuous measure, where higher scores indicate higher depressive symptoms or as an ordinal indicator of possible depression using cut-off points previously used in literature [2].

***Depression, anxiety, stress (DASS-42).*** Depression, anxiety and stress were additionally measured using the Depression Anxiety Stress Scale-42 (DASS-42) by Lovibond & Lovibond. The 42-item scale has been validated for clinical and non-clinical samples and has been proven to be a reliable screening tool for symptoms of depression, anxiety and stress. Prior studies have demonstrated high internal consistency and convergent and discriminant validity of the DASS. The questionnaire is divided into three scales (one for each state) each containing 14 items. Items are scored on a four-point Likert scale ranging from 0 (did not apply to me at all) to 3 (applied to me very much) measuring the extent to which each state was experienced over the past week. Scores for each of the three scales are determined by summing up the scores and categorized into: depression 0-9 (normal), 10-20 (mild-moderate), >21 (severe-very severe); anxiety 0-7 (normal), 8-14 (mild-moderate), >15 (severe-very severe); stress 0-14 (normal); 15-25 (mild-moderate), >26 (severe-very severe) [3].

***Somatic symptoms (PHQ-15).*** The Patient Health Questionnaire-15 (PHQ-15) is a 15-item, somatic symptom scale derived from the full Patient-Health-Questionnaire to measure the severity of somatization in patients. Patients were asked to indicate the severity of 15 physical symptoms they may have experienced during the past four weeks on a three-point scale 0 (“not bothered at all”) to 2 (“bothered a lot”). The total PHQ-15 score was obtained by summing the scores of each item and classified as minimal (0-4); mild (5-9); moderate (10-14); and high (15-30) with higher scores indicating greater severity of somatic symptoms. Prorated scores were calculated if there were unanswered items on the measure [4].

***Quality of life (SF-12-PCS; SF-12-MCS).*** Quality of life was assessed using the 12-item Short Form Health Survey (SF-12), an alternative to the SF-36 that measures overall health status. The SF-12 is comprised of two components, the Mental Component Summary (MCS) score and the Physical Component Summary (PCS) score. The SF-12 consists of 12 items including mental health items (vitality, role-emotional, social function, and mental health) and physical health items (physical functioning, physical role, bodily pain). The possible scores of the 12 questions range from 0-100, in which lower scores reflect lower levels of health and higher scores reflect higher levels of health. The MCS and PCS scores were standardized based on data of a US general population using a mean score of 50 and a standard deviation (SD) of 10 [5].

***Post-traumatic stress disorder (PTSD).*** The Post-Traumatic Stress Disorder Checklist (PTSD-PCL-S) is a 17-item scale used to assess PTSD symptoms based on the DSM-IV criteria. Each item in the checklist is scored from 1 (not at all) to 5 (extremely), indicating the extent to which the patient has been bothered by the symptom in the past month. A total symptom severity score is calculated. The range of the total score is divided into four categories of PTSD symptom severity: “little” (17-29), “some” 28-29 “moderate-moderately high” (30-44), and ”high” (45-85) [6].

***Social Support (ESSI).*** Social support was assessed using the seven-item ENRICHD (Enhancing Recovery in Coronary Heart) Social Support Instrument (ESSI) comprised from the Medical Outcomes Survey (MOS) and assesses for four components of social support including emotional, instrumental informational and appraisal. The ESSI has been used in previous studies on cardiac populations with post-MI and CAD. It has demonstrated high internal consistency, reliability, and good convergence. Items on the ESSI are summed up for a total score with item categories ranging from 1 (none of the time) to 5 (all of the time). Patients who score ≤18 on the scale are considered to have low social support and meet the ENRICHED eligibility criteria [7, 8].

***Self-esteem (SE).***The Single-Item Self-Esteem Scale is a one-item scale developed as an alternative of the Rosenburg Self-Esteem scale which is used to measure self-esteem. It is based on a seven-point Likert scale from 1 (not very true of me) to 7 (very true of me) [9].

***Resilience (RS-14).*** Resilience Scale-14 (RS-14) is a 14-item questionnaire that assesses resilience in a general population. The RS-14 is comprised of five characteristics of the resilience core, including purpose, perseverance, self-reliance, equanimity, and authenticity. Items of the RS-14 are scored on a seven-point Likert scale from 1 (strongly disagree) to 7 (strongly agree). Total scores range from 14 to 98, and are categorized as very low (14-56); low (57-64); on the low end (65-73); moderate (74-81); moderately high (82-90) and high (91-98) resilience levels [10].

**References (Additional file 1)**

1. Hare DL, Davis CR: **Cardiac Depression Scale: validation of a new depression scale for cardiac patients.** J Psychosom Res 1996, **40:**379-386.

2. Shi WY, Stewart AG, Hare DL: **Major depression in cardiac patients is accurately assessed using the cardiac depression scale.** Psychother Psychosom 2010, **79:**391-392.

3. Lovibond SH LP: **Manual for the depression anxiety stress scales.** 2 edition Sydney Psychology Foundation 1995.

4. Kroenke K, Spitzer RL, Williams JB: **The PHQ-15: validity of a new measure for evaluating the severity of somatic symptoms.** Psychosom Med 2002, **64:**258-266.

5. Ware J, Jr., Kosinski M, Keller SD: **A 12-Item Short-Form Health Survey: construction of scales and preliminary tests of reliability and validity.** Med Care 1996, **34:**220-233.

6. Blanchard EB, Jones-Alexander J, Buckley TC, Forneris CA: **Psychometric properties of the PTSD Checklist (PCL).** Behav Res Ther 1996, **34:**669-673.

7. **Enhancing recovery in coronary heart disease patients (ENRICHD): study design and methods. The ENRICHD investigators.** Am Heart J 2000, **139:**1-9.

8. **Enhancing Recovery in Coronary Heart Disease (ENRICHD) study intervention: rationale and design.** Psychosom Med 2001, **63:**747-755.

9. Lett HS, Blumenthal JA, Babyak MA, Sherwood A, Strauman T, Robins C, Newman MF: **Depression as a risk factor for coronary artery disease: evidence, mechanisms, and treatment.** Psychosom Med 2004, **66:**305-315.

10. Wagnild GM, Guinn PE: The Resilience Scale User's Guide: For the U.S. English Version of The Resilience Scale TM and the 14-Item Resilience Scale TM (RS-14 TM). Resilience Center; 2009.
