# Supplementary material for: Depression and anxiety symptoms in cardiac patients: a cross-sectional hospital-based study in a Palestinian population
Source: BMC Public Health. 2019 Feb 26;19:232. doi: 10.1186/s12889-019-6561-3 (PMC6390372; doi:10.1186/s12889-019-6561-3)
Supplement: Supplementary file 2 — Table S2. Socio-demographic, clinical, psychosocial, lifestyle factors by STRESS status, (n = 1022). (DOCX 24 kb) [file 12889_2019_6561_MOESM2_ESM.docx]

**Table S2** Socio-demographic, clinical, psychosocial, lifestyle factors by STRESS status,

(n=1022)

| **Variable** | **Stress (DASS-stress)** | | |
| --- | --- | --- | --- |
|  | No  n= 370  (%)^†^ | Yes  n=652 (%)^††^ | *P* value |
| **Socio-demographic factors** | | | |
| Age, mean (SD)  Gender  Female  Male  Marital status  Married  Not married  Residence  City  Village  Camp  Education degree  No HS diploma  HS diploma  College degree  Occupation  Professional  Non-professional  Unemployed  Retired  House wife | 60.3±10.1*  36.0  36.3  36.3  35.4  40.3  33.4  28.0  35.2  34.6  42.8  33.0  41.0  36.2  29.6  29.8 | 58.3±10.1*  64.0  63.7  63.7  64.6  59.7  66.6  72.0  64.8  65.4  57.2  67.0  59.0  63.8  70.4  70.2 | **0.002**  0.944  0.866  **0.027**  0.171  0.172 |
| ***Clinical factors*** | | | |
| Cardiac diagnosis  CAD  MI  Angina  Other  Previous cardiac diagnosis  Yes  No  Years with cardiac disease  ≤1 year  2-9 years  ≥10 years  Cardiac treatment (at admission)  CATH/stent  CATH/CABG  CATH/other & unknown  Co-morbidities  None  One  Two or more  Medications  None  1-2  3-4  Somatic symptoms (PHQ-15)  Minimal  Low  Medium  High  Family history  Yes  No  QoL, (SF-12-PCS score), mean (SD) | 28.1  38.4  51.2  30.8  34.2  40.4  39.7  32.6  27.3  37.1  35.0  35.5  42.5  34.0  33.3  33.3  30.9  37.8  56.0  50.2  34.7  23.7  38.0  35.0  40.0±12.3* | 71.9  61.6  48.8  69.2  65.8  59.6  60.3  67.4  72.7  62.9  65.0  64.5  57.5  66.0  66.7  66.7  69.1  62.2  44.0  49.8  65.3  76.3  62.0  65.0  36.3±12.2* | **<0.001**  0.055  **0.009**  0.826  **0.027**  0.209  **<0.001**  0.333  **<0.001** |
| ***Psychosocial factors*** | | | |
| PTSD (PTSD-PCL-S)  Minimal  Some  Moderate  High  Social support (ESSI)  Low  High  Resilience (RS-14)  Very low  Low  Low-end  Moderate  Moderately-high  High  Self-esteem (SE) score, mean (SD)  QoL, (SF-12-MCS score), mean (SD) | 52.9  17.9  10.3  6.3  36.0  36.3  14.1  23.3  38.2  43.2  41.2  33.7  6.0±1.2*  44.6±12.4* | 47.1  82.1  89.7  93.7  64.0  63.7  85.9  76.7  61.8  56.8  58.8  66.3  5.7±1.5*  37.0±12.9* | **<0.001**  0.915  **<0.001**  **<0.001**  **<0.001** |
| ***Lifestyle factors*** | | | |
| Smoking status  Never  Former  Current  Currently on diet  Yes  No  Fat consumption  Low  Medium  High  Vegetable & fruit consumption  Low  Medium  High  Alcohol use  Yes  No  Physical activity  None  Not daily  Daily  BMI  Underweight  Normal weight  Overweight  Obese | 37.6  35.3  35.4  38.2  35.8  35.4  38.7  34.2  32.3  37.1  36.3  16.3  37.3  28.8  39.7  39.9  0.0  32.7  40.5  33.7 | 62.4  64.7  64.6  61.8  64.2  64.6  61.3  65.8  67.7  62.9  63.7  83.7  62.7  71.2  60.3  60.1  100.0  67.3  59.5  66.3 | 0.777  0.546  0.506  0.676  **0.003**  **0.002**  **0.049** |

*Note.* Bivariate analysis was performed using chi-squared test to assess the association of depression with factors of the four predictor blocks. Analysis was adjusted for hospital site. HS= high school; MI= myocardial infarction; CAD= coronary artery disease; CATH= catheterization; CABG= coronary artery bypass graft; CVD= cardiovascular disease; PHQ-15= Patient Health Questionnaire; PCS= Physical Component Summary; QoL= quality of life; SD= standard deviation; PTSD=post-traumatic stress disorder; PTSD-PCL-S=Post-Traumatic Stress Disorder Checklist; ESSI= ENRICHD Social Support Instrument; RS-14= Resilience Scale-14; MCS=Mental Component Summary; BMI= body mass index *=Wilcoxon rank sum test; † Scores 0-14 (no stress); †† Scores 15-42 (mild, moderate, severe, very severe stress); *P* values in bold are significant at p <0.05.
