# Supplementary material for: Depression and anxiety symptoms in cardiac patients: a cross-sectional hospital-based study in a Palestinian population
Source: BMC Public Health. 2019 Feb 26;19:232. doi: 10.1186/s12889-019-6561-3 (PMC6390372; doi:10.1186/s12889-019-6561-3)
Supplement: Supplementary file 3 — Table S1. Socio-demographic, clinical, psychosocial, lifestyle factors by DEPRESSION and ANXIETY status, (n = 1022). (DOCX 24 kb) [file 12889_2019_6561_MOESM3_ESM.docx]

**Table S1** Socio-demographic, clinical, psychosocial, lifestyle factors by DEPRESSION and ANXIETY status, (n=1022)

| **Variable** | **Depression (CDS)** | | | **Anxiety (DASS-anxiety)** | | |
| --- | --- | --- | --- | --- | --- | --- |
|  | NO  n=218  (%)^†^ | YES  n=804  (%)^††^ | *P* value | NO  n=408  (%)^†††^ | YES  n=614  (%)^††††^ | *P* value |
| ***Socio-demographic factors*** | | | | | | |
| Age, mean (SD)  Gender  Female  Male  Marital status  Married  Not married  Residence  City  Village  Camp  Education degree  No HS diploma  HS diploma  College degree  Occupation  Professional  Non-professional  Unemployed  Retired  House wife | 58.9±10.6*  15.1  23.6  20.6  28.1  23.0  20.9  13.3  19.0  22.1  28.9  22.5  25.1  15.9  32.1  17.0 | 59±10.0*  84.9  76.4  79.4  71.9  77.0  79.1  86.7  81.0  77.9  71.1  77.5  74.9  84.1  67.9  83.0 | 0.560  **0.003**  0.088  0.158  **0.024**  **0.004** | 59.2±9.8*  27.6  44.4  40.7  32.3  40.1  40.2  37.3  36.8  42.5  47.2  45.0  47.6  31.0  49.4  23.4 | 58.8±10.3*  72.4  55.6  59.3  67.7  59.9  59.8  62.7  63.2  57.4  52.8  55.0  52.4  69.0  50.6  76.6 | 0.663  **<0.001**  0.109  0.893  **0.036**  **<0.001** |
| ***Clinical factors*** | | | | | | |
| Cardiac diagnosis  CAD  MI  Angina  Other  Previous cardiac diagnosis  Yes  No  Years with cardiac disease  ≤1 year  2-9 years  ≥10 years  Cardiac treatment (at admission)  CATH/stent  CATH/CABG  CATH/other & unknown  Co-morbidities  None  One  Two or more  Medications  None  1-2  3-4  Somatic symptoms (PHQ-15)  Minimal  Low  Medium  High  Family history  Yes  No  QoL, (SF-12-PCS score), mean (SD) | 24.9  21.7  17.3  15.8  19.0  26.2  24.0  18.0  15.1  24.3  17.9  18.1  28.4  21.8  16.0  28.8  19.7  20.3  40.7  36.9  18.1  9.3  20.8  21.7  42.7±12.7* | 75.1  78.3  82.7  84.2  81.0  73.8  76.0  82.0  84.9  75.7  82.1  81.9  71.6  78.2  84.0  71.2  80.3  79.7  59.3  63.1  81.9  90.7  79.2  78.3  36.3±11.9* | 0.101  **0.008**  **0.022**  **0.048**  **<0.001**  0.080  **<0.001**  0.752  **<0.001** | 39.5  37.9  54.3  28.3  33.5  53.3  43.3  34.1  35.2  44.2  38.2  31.4  48.2  40.9  33.3  50.0  34.9  39.2  72.5  61.4  39.2  18.9  42.9  37.9  43.1±11.8* | 60.5  62.1  45.7  71.7  66.5  46.7  56.7  65.9  64.8  55.8  60.8  68.6  51.8  59.1  66.7  50.0  65.1  60.8  27.5  38.6  60.8  81.1  57.1  62.1  34.0±11.3* | **<0.001**  **<0.001**  **0.020**  **0.003**  **<0.001**  **0.025**  **<0.001**  0.114  **<0.001** |
| ***Psychosocial factors*** | | | | | | |
| PTSD (PTSD-PCL-S)  Minimal  Some  Moderate  High  Social support (ESSI)  Low  High  Resilience (RS-14)  Very low  Low  Low-end  Moderate  Moderately-high  High  Self-esteem (SE) score, mean (SD)  QoL, (SF-12-MCS score), mean (SD) | 29.0  14.7  10.7  0.0  19.2  22.5  9.8  9.7  19.6  22.2  26.6  31.5  6.2±1.0*  46.3±12.5* | 71.0  85.3  89.3  100.0  80.8  77.5  90.2  90.3  80.4  77.8  73.4  68.5  5.7±1.5*  37.9±12.9* | **<0.001**  **<0.001**  **<0.001**  **<0.001**  **<0.001** | 53.5  25.3  20.2  9.5  34.3  43.0  7.6  14.6  31.4  42.4  58.8  56.5  6.3±0.9*  44.4±12.8* | 46.5  74.7  79.8  90.5  65.7  57.0  92.4  85.4  68.6  57.6  41.2  43.5  5.4±1.5*  36.7±12.6* | **<0.001**  **0.007**  **<0.001**  **<0.001**  **<0.001** |
| ***Lifestyle factors*** | | | | | | |
| Smoking status  Never  Former  Current  Currently on diet  Yes  No  Fat consumption  Low  Medium  High  Vegetable & fruit consumption  Low  Medium  High  Alcohol use  Yes  No  Physical activity  None  Not daily  Daily  BMI  Underweight  Normal weight  Overweight  Obese | 22.3  19.4  20.9  27.1  20.2  22.1  21.7  19.2  17.7  19.8  22.9  22.5  21.2  12.5  19.1  28.2  25.0  21.1  25.1  17.4 | 77.7  80.6  79.1  72.9  79.8  77.9  78.3  80.8  82.3  80.2  77.1  77.5  78.8  87.5  81.9  71.8  75.0  78.9  74.9  82.6 | 0.701  **0.046**  0.674  0.340  0.840  **<0.001**  <0.063 | 42.6  49.4  31.9  33.5  41.1  42.9  37.2  37.4  41.2  45.0  36.5  34.7  40.3  31.0  48.7  42.5  0.0  36.2  47.1  34.4 | 57.4  50.6  68.1  66.5  58.9  57.1  62.8  62.6  58.8  55.0  63.5  65.3  59.7  69.0  51.3  57.5  100.0  63.8  52.9  65.6 | **<0.001**  0.062  0.184  **0.035**  0.434  **<0.001**  **<0.001** |

*Note.* Bivariate analysis was performed using chi-squared test to assess the association of depression with factors of the four predictor blocks. Analysis was adjusted for hospital site. HS= high school; MI= myocardial infarction; CAD= coronary artery disease; CATH= catheterization; CABG= coronary artery bypass graft; CVD= cardiovascular disease; PHQ-15= Patient Health Questionnaire-15; PCS= Physical Component Summary; QoL= quality of life; SD= standard deviation; PTSD= post-traumatic stress disorder; PTSD-PCL-S=Post-Traumatic Stress Disorder Checklist; ESSI= ENRICHD Social Support Instrument; RS-14= Resilience

Scale-14; MCS= Mental Component Summary; BMI= body mass index *= Wilcoxon rank sum test; † Scores 26-89 (no depressive symptoms); †† Scores ≥90 (depressive symptoms); ††† Scores of 0-6 (no anxiety); †††† Scores ≥7 (mild, moderate, severe, very severe anxiety); *P* values in bold are significant at p <0.05.
